# Activation of endothelial damage by TNFα and IFNγ in ischemia/reperfusion injury and systemic inflammation

**DOI:** 10.1186/cc9836

**Published:** 2011-03-11

**Authors:** A Golovkin, E Grigoryev, V Matveeva, G Plotnikov, D Shukevich

**Affiliations:** 1 Institute of Complex Problems of Cardiovascular Diseases, Kemerovo, Russia

## Introduction

The objective was to verify the possibility of endothelial damage induced by cytokines in ischemia/reperfusion (extracorporeal circulation).

## Methods

Forty-one patients were included in the study. All patients diagnosed with coronary artery disease were operated on in the amount of coronary artery bypass grafting under normothermic cardiopulmonary bypass nonpulsed (CB) with cold blood cardioplegia. Systemic inflammatory response (SIRS) was defined as: SIRS I - 57%, SIRS II - 24%, SIRS III - 19%. Ischemia/reperfusion was confirmed by oxygen status and lactate of arterial and mixed venous blood (StatProfile). We investigated by enzyme immunoassay analysis (ELISA): soluble triggering receptor expressed on myeloid cells (sTREM-1), TNFα, IFNγ, soluble vascular cell adhesion molecules (sVCAM-1), soluble intercellular adhesion molecule (sICAM-1), and soluble platelet/endothelial cell adhesion molecule (sPECAM-1); sets from Bender Medsystems and CanAg. Data are presented as mean ± standard deviation.

## Results

In all patients was reported a decrease in the content of TNFα, and IFNγ (first point - before the extracorporeal circulation, second point - after). However, after the separation of patients according to severity of SIRS, a group of patients with the definition of the three signs of the cellular adhesion molecules (which corresponds to the most severe course of clinical and laboratory manifestations of systemic inflammation) recorded an increase in the concentration of TNFα, as well as sVCAM-1 (4.45 ± 0.9 vs. 8.9 ± 0.9 pg/ml), and sPECAM-1 (3.4 ± 0.9 vs. 6.7 ± 0.9 pg/ml). The level of sICAM-1 increased both in the general population and separately in groups of patients with different levels of expression of the cellular adhesion molecules. Similar results were obtained for the level of sTREM-1. A direct correlation was observed between the level of leading cytokines, the level of sTREM-1 and the level of cell adhesion molecules.

## Conclusions

There is endothelial damage, activated by cytokines, reaching the highest value at SIRS III during ischemia/reperfusion and systemic inflammation.

